# Contamination and health risk assessment of heavy metals in soil surrounding an electroplating factory in JiaXing, China

**DOI:** 10.1038/s41598-024-54620-w

**Published:** 2024-02-19

**Authors:** Tingting Liu, Zhen Wang

**Affiliations:** 1 Institute of Environmental Engineering, Department of Ecological Health, Hangzhou Vocational & Technical College, Hangzhou, 310018 People’s Republic of China; 2 Key Laboratory of Environmental Pollution Control Technology Research of Hejiang Province, Eco-Environmental Science Research & Design Institute of Zhejiang Province, Hangzhou, 310007 People’s Republic of China

**Keywords:** Electroplating factory, Heavy metal contamination, Index of geoaccumulation, Human health risk assessment, Environmental sciences, Environmental social sciences, Health care

## Abstract

A total of 30 samples from the downwind direction of a certain electroplating company in Jiaxing were collected in layers to analyze their heavy metal content. The soil risk assessment was conducted from the perspective of ecological and human health risks using the ground accumulation index method and human health risk assessment method. The results showed that in all samples, cadmium and arsenic far exceeded the soil background values, with an average exceeding multiple of 14.31 and 64.42, respectively, and a exceeding rate of 100%. After evaluation by the ground accumulation index, among these six heavy metals, arsenic and cadmium belong to extremely serious pollution levels. The human health risk assessment of electroplating plants found that in the exposure risk assessment, the ingestion value was much greater than the harm caused by breathing and skin, and the maximum exposure damage value of arsenic to children and adults was 4.17 × 10^–3^, among the carcinogenic risks, the risk brought by consumption is much greater than the respiratory and skin carcinogenic risk index, with the highest value score of 3.37 for cadmium, arsenic, and zinc carcinogenic risks 3.37 × 10^–6^, 2.42 × 10^–3^, 1.10 × 10^–4^.

## Introduction

The metal surface treatment (electroplating) industry is a pillar industry in the South Lake region. The "three wastes" in the traditional metal surface treatment industry sometimes leak, causing serious pollution to the surrounding environment^1^. However, the electroplating industry is an indispensable part of industries such as automotive parts, electronics, and hardware processing. The large amount of metal wastewater discharged from electroplating wastewater, as well as dust and various inorganic pollutant particles containing metal pollutants such as As, Cu, Pb, Cd, Ni, etc. generated during the electroplating process, settle into the soil with water and atmosphere, posing a huge threat to the safety of surrounding soil and agricultural products. Electroplating wastewater contains a large amount of chromium ions, which settle into the soil and enter the human body along with the soil food chain. If human ingests a large amount of trivalent chromium atoms(the maximum concentration of trivalent chromium is 120 µM)^2^, it will lead to chronic oxidative damage. Oxidative damage will lead to some disease such as diabetes, hypertension, tumor or other hyperplasia^3^. Hexavalent chromium is more harmful to human body, and may also cause cancer in serious cases. Zinc contamination caused by processes such as cyanide copper plating, zinc plating, alloy plating, and imitation gold plating can have an impact on children's growth, development, and physical health^4^, leading to severe kidney failure. At the same time, the solid waste in the electroplating process is mainly sludge from chemical disposal, which contains a large amount of different toxic metal substances^5^, which also poses a serious burden on the soil. The reduction in yield from the surrounding crops reflect the damage caused by heavy metal contamination to nearby soils.

At present, although some scholars have investigated and studied the content of heavy metals in the soil of typical factory areas in Jiaxing City^6–8^, the number of survey points is very small, the survey has a long history, and most of them are limited to a certain element, while the comprehensive and systematic investigation and study of heavy metal pollution in the soil of key electroplating areas in Jiaxing City has not yet been reported. In view of this, this study possess a large number of measured data, this paper systematically explored the status quo of heavy metal pollution of Jiaxing electroplating plant on surrounding agricultural land soil and carried out risk assessment, in order to provide basis for early warning of soil environmental quality in Jiaxing. Starting from the evaluation of the ecological and environmental effects of heavy metal pollution in soil, this article uses the ground accumulation index and potential ecological hazard index of heavy metals in soil as pollution evaluation indicators to evaluate the level of heavy metal pollution in agricultural soil in the study area. It has guiding significance for the control of heavy metals in farmland soil in Jiaxing City.

## Materials and methods

### Overview of the study area

A plating company in Jiaxing City is located in Zhuangshi Village, Fengqiao Town, Nanhu District, which is in the center of the Yangtze River Delta metropolitan area, 25 km away from the downtown area of Jiaxing City, and within one hour, you can reach Shanghai, Hangzhou and Suzhou. The transportation is very convenient. Mainly engaged in metal processing, die-casting processing and other businesses, the factory area of 2000 square meters, specializing in various metal products enterprises to provide nickel-plated, chrome, and other metal products processing services. The factory contributes to the local economy at the same time, but also has a great impact on the surrounding environment. The region has a typical subtropical monsoon climate, with high temperature and rain in summer and low temperature and rain in winter. The soil in the area is mainly dominated by tidal red soil, tidal red soil and clay soil, which has good water storage performance and permeability, and is not susceptible to drought and flooding, but has a certain salt content. In order to rationally utilize land resources and protect human health, this study selects seven typical heavy metals (Cr, As, Pb, Ni, Cu, Zn, Cd) for pollution evaluation and health risk evaluation based on the health of the residents as an orientation.

### Sample collection and determination

#### Soil sampling and pretreatment

**Figure 1:**
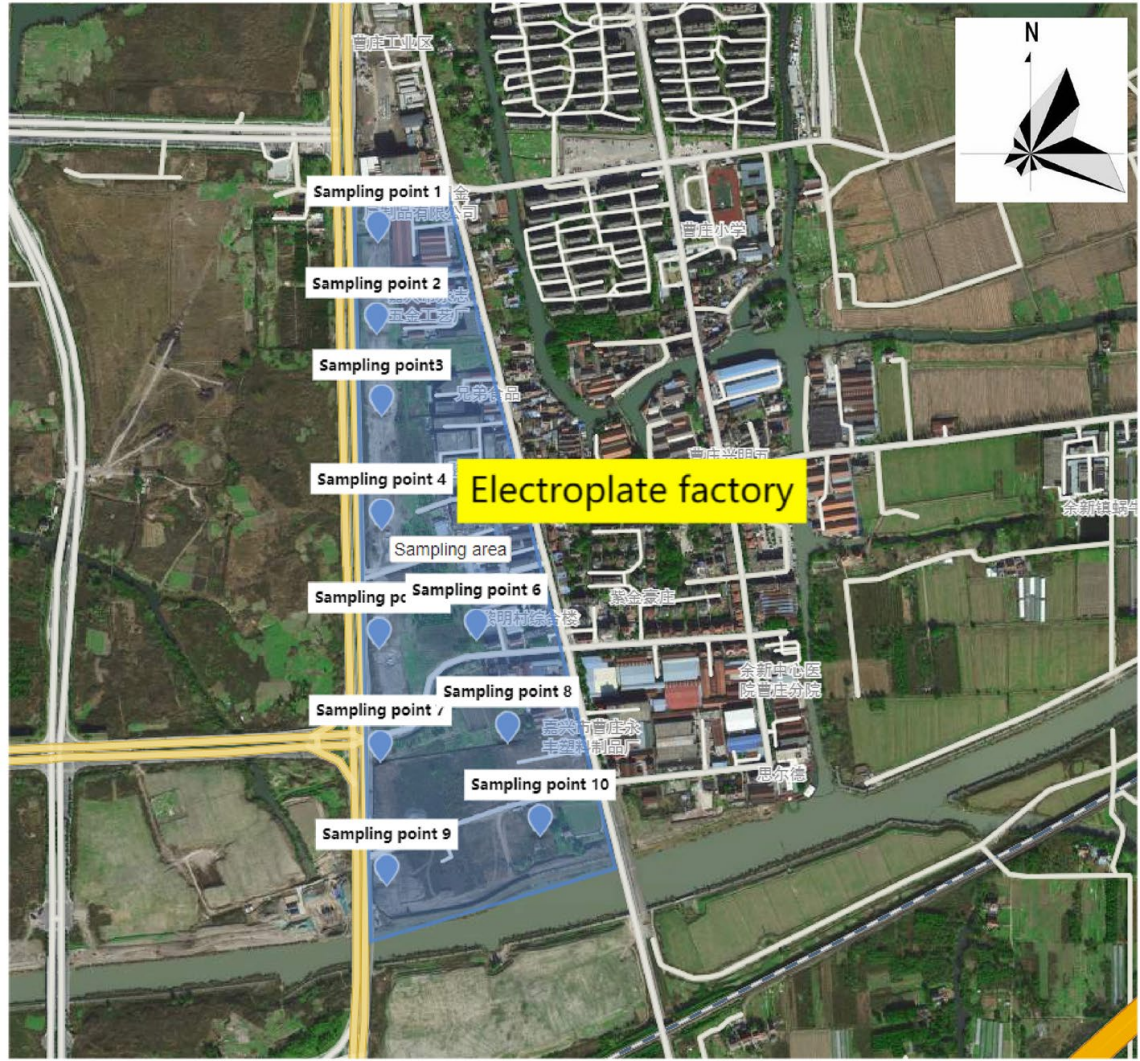
Sampling Distribution Map (This figure were generated used by the software (Dingmap, the URL link is https://dm.dingmap.com/home/map?id=6fdbbddf6d2240e5a0a72d187374eacc).

In this study, the grid method was used to arrange sampling points to investigate the pollution status of the topsoil in this block, and to find out the distribution of heavy metal pollution concentration in this key block. At the same time, further investigation was conducted on the depth of heavy metal pollution in the soil through more cross-sectional sampling. Specifically, in the southeast downwind area along the perennial wind direction of the factory, at a distance of 100 m from the edge of the factory. Soil samples were collected from top to bottom according to the vertical depth of 0–10, 10–20, 20–30 cm respectively, totaling 30, the sampling diagram is shown in Fig. 1, the Fig. 1 were generated used by the software(Dingmap the URL link is https://dm.dingmap.com/home/map?id=6fdbbddf6d2240e5a0a72d187374eacc). First I find the sampling area, Then draw with polygons, the sampling area is then shaded. After that, another layer is created, the sampling points are labeled, and finally multiple layers are displayed on the same map.When sampling, 1–2 kg was collected and put into the sampling bag, labeled with the sample information and number. The samples were brought back to the laboratory and placed in a ventilated place to dry naturally, then ground and passed through a nylon sieve of 100 m mesh and digested.

#### Soil testing

The soil samples were air-dried at room temperature in a cool and ventilated place, and plant roots, stones and other debris were removed from the soil samples, which were then ground in an agate mortar and sieved through a 100-mesh sieve and put into a sample bag to be tested. 0.2 g of soil sample was weighed for heavy metal content. The soil sample is pre-treated with 5 mL HNO_3_, 5 mL HF, and 3 mL HClO_4_ dissolved at high temperature and digested in a microwave digestion device. Then, Inductively coupled plasma Spectrometer (ICP-MS) was used for the total analysis of soil elements^9^. The agents used in the experiments were of superior purity, and all the experimental supplies were soaked in ten percent dilute nitric acid overnight and washed in ultrapure water. Blanks and parallel samples were used as controls throughout. The recoveries of each metal were within the allowable range of the national standard reference material. During the measurement process, quality control was performed by adding national standard substances and setting up parallel samples.

### Evaluation method

#### Index of geo-accumulation method

This method was proposed by the German scientist Muller and is used to quantitatively evaluate the degree of heavy metal pollution in sediments^10,11^. In addition to human pollution factors and environmental geochemical background values, the factors of background value changes caused by natural Diagenesis are also considered in the evaluation process^12,13^。

#### Index of geo-accumulation

$$I_{geo} = \log_{2} \left[ {\frac{{C_{s}^{i} }}{{K \times C_{n}^{i} }}} \right]$$

In the equation:

$C_{s}^{i}$ Is the content of element i in sediment;

$C_{n}^{i}$ Is the geochemical background value of this element in the sediment^14–17^;

K is a coefficient taken to consider the possible changes in background values caused by differences in rocks in different regions (usually taken as 1.5).

The calculation results are divided into pollution levels according to the evaluation criteria of index of geo-accumulation (Table 1)^14^。

**Table 1:** Criteria for index of geo-accumulation(*I*_*geo*_)^16^.

Project	Grade	Pollution level
*I*_*geo*_	0	Non-pollution
*0* < *I*_*geo*_*≦1*	1	Mild poisoning pollution
*1* < *I*_*geo*_*≦2*	2	Moderate pollution
*2* < *I*_*geo*_*≦3*	3	Moderate to strong pollution
*3* < *I*_*geo*_*≦4*	4	Strong pollution
*4* < *I*_*geo*_*≦5*	5	Strong pollution—extremely severe pollution
*5* < *I*_*geo*_*≦10*	6	Extreme pollution

### Human health risk assessment method

Heavy metals in soil pose a threat to human health through three main pathways: direct inhalation of soil dust into the air through oral and nasal breathing; Transferring fruits, vegetables, grains, etc. in the food chain through contaminated soil; Direct skin contact contaminates soils with heavy metals^18,19^. The industrial process of surface treatment of this metal generates a large amount of smoke and dust, which is close to farmland. All three pathways mentioned above may become the main pathways endangering human health. Therefore, in this study, the non carcinogenic risk assessment and carcinogenic risk assessment of heavy metals on human health are all included in the model^18,19^。

### Exposure assessment calculation

The amount of pollution ingested by inhaling soil dust through respiration:

$$EDI_{breathe} { = }\frac{{{\text{CS}} \times {\text{IR}}_{air} \times {\text{EF}} \times {\text{ED}}}}{{{\text{PEF}} \times {\text{BW}} \times {\text{AT}}}}$$

Amount of pollution ingested through direct skin contact with soil:

$$EDI_{skin} { = }\frac{{{\text{CS}} \times {\text{SA}} \times {\text{AF}} \times {\text{ABS}} \times {\text{EF}} \times {\text{ED}}}}{{{\text{BW}} \times {\text{AT}}}} \times 10^{{{ - }6}}$$

Direct oral intake of soil pollution:

$$EDI_{mouth} { = }\frac{{{\text{CS}} \times {\text{IR}}_{soil} \times {\text{EF}} \times {\text{ED}}}}{{{\text{BW}} \times {\text{AT}}}} \times 10^{{{ - }6}}$$

Total exposure:

$$EDI_{total} { = }EDI_{breathe} { + }EDI_{skin} { + }EDI_{mouth}$$

In the formula, *EDI*_breathe_, *EDI*_*s*kin_, *EDI*_mouth_ refer to the total amount of pollutants in soil ingested through respiratory inhalation, skin contact, direct oral intake, and the above three pathways, mg/kg per day; CS is the heavy metal content in the soil, mg/kg; IR soil refers to the soil intake rate, m^3^/d; IR air is the intake rate of air, m3/d;. PEF is the soil dust generation factor m^3^/kg; SA is the skin contact surface area, cm^2^/d; AF is the skin's adsorption coefficient, mg/cm^2^; ABS is the skin absorption rate,%; EF is the exposure frequency, d/a; ED is the exposure period/a; BW is the body mass, kg; AT is the average action time, d.

The environmental risk assessment standards for adults and children are quite different when conducting Exposure assessment. According to China's site environmental assessment guidelines, the USEPA health risk assessment method and the actual research conclusions at home and abroad in recent years^20–27^, the values of each Exposure assessment parameter in this assessment are shown in Table 2

**Table 2:** The values of exposure parameter.

Parameter	Value	
	Children	Adult
IRsoil/(m^3^/d)	200	100
IRair/(m^3^/d)	7.5	15
EF/(d/a)	350	350
ED/a	6	30
SA/(cm^2^/d)	1600	5000
AF/(mg/cm^2^)	0.07	0.2
ABS	0.001	0.001
PEF/(m^3^/kg)	1.36 × 10^9^	1.36 × 10^9^
BW/kg	15.9	55.9
AT/d	Carcinogenic70 × 365	noncarcinogenic30 × 365

### Toxicity assessment and risk characterization

Toxicity assessment is the estimation of the relationship between population exposure to pollutants and the likelihood of negative effects^5,28^. Among the six heavy metals studied in this article, Cu, Pb, Cd, and Ni all have non carcinogenic health risks, among which Pb, Cd, and Ni also have carcinogenic risks^5,28^. The non carcinogenic and carcinogenic toxicity parameters of four heavy metals, Cu, Pb, Cd, and Ni, are shown in Table 3. The non carcinogenic toxicity parameters are the reference dose (RfDj) of heavy metals under each exposure pathway, and the carcinogenic effect reference number (SF) is the carcinogenic slope factor of Cd and Ni.

**Table 3:** RfD and SF of heavy metals for different exposure routes^29,30^.

Heavymetal	RfD mouth/	RfD breath/	RfD skin/	SF/
	[mg/(kg d)]	[mg/(kg d)]	[mg/(kg d)]	[(kg d)/mg]
Cd	1 × 10^–2^	1 × 10^–3^	1 × 10^–5^	3.8 × 10^–1^
Ni	2 × 10^–2^	2.06 × 10^–2^	5.4 × 10^–3^	8.4 × 10^–1^
As	3 × 10^–4^	3.83 × 10^–6^	3 × 10^–4^	1.5 × 10^0^
Zn	3 × 10^–1^	0	3 × 10^–1^	

Each exposure route has carcinogenic and non carcinogenic risks. The non carcinogenic risk level can be calculated by dividing the daily exposure of heavy metals by the chronic reference dose of three routes, namely, oral, skin and respiratory. The calculation formula is as follows:

$$HQ_{i} = \frac{{EDI_{j} }}{{RfD_{j} }}\;\;\;\;\;\;\;\;\;HI = \sum {HQ}_{i}$$

where HI is the total non carcinogenic risk level of soil heavy metals under three exposure routes: oral, respiratory and skin contact; HQi represents the non carcinogenic risk level of different intake pathways; EDIj is the average daily intake of pollutants from different pathways, mg/(kg'd), while RfDj is the chronic reference dose for each pathway, mg/(kg. d) (see Table 3). When HQi < 1 or HI < 1, there is no significant non carcinogenic health risk; When HQi > 1 or HI > 1, it indicates a non carcinogenic health risk, and the higher the value, the more severe the non carcinogenic health risk.

The level of cancer risk is calculated by multiplying the average daily intake to the entire life cycle by the slope coefficient of carcinogenicity through oral, skin, or respiratory inhalation. The calculation formula is:

$$\begin{aligned} Risk_{i} = & EDI_{i} \times SF_{i} \\ (Risk)_{T} = & \sum {Risk_{i} } \\ \end{aligned}$$

In the formula, Risk_i_ is the carcinogenic risk index of soil heavy metals under different pathways; (Risk)_T_ is the comprehensive risk index of heavy metal carcinogenesis in soil; EDI is the average daily intake of different pollutants, mg/(kg'd); SF_i_ is the slope coefficient of cancer risk for various pathways, (kg'd)/mg (see Table 3). Risk is the carcinogenic health index, usually represented by a certain number of recognized cancer patients. The acceptable risk value of Carcinogen defined by the U.S. Environmental Protection Agency is 10^–4^–10^–6^, the risk of cancer incidence in a lifetime exceeds the normal value. When risk < 1 × 10^–6^ h, it is considered that there is no risk of cancer; When Risk > 1 × 10^−4^ h, it is considered that there is a risk of cancer; When 1 × 10−^6^ ≤ Risk ≤ 1 × 10^−4^ h, the risk of cancer is considered within an acceptable range.

### Ecological hazard index

The ecological hazard index not only considers the content of heavy metals in soil based on their properties and environmental behavior characteristics, but also links the ecological and environmental effects of heavy metals with toxicology. It adopts a comparable and equivalent attribute index grading method for evaluation.

The formula is:

$$C{}_{{\text{f}}}^{{\text{i}}} = C{}_{e}^{{\text{i}}} /C{}_{{\text{n}}}^{{\text{i}}} ,\;E{}_{{\text{r}}}^{{\text{i}}} = C{}_{{\text{f}}}^{{\text{i}}} \times T{}_{{\text{r}}}^{{\text{i}}} ,\;RI = \sum\limits_{{{\text{i}} = 1}}^{{\text{n}}} {E{}_{{\text{r}}}^{{\text{i}}} } = \sum\limits_{{{\text{i}} = 1}}^{{\text{n}}} {T{}_{{\text{r}}}^{{\text{i}}} } \times C{}_{{\text{f}}}^{{\text{i}}} = \sum\limits_{{{\text{i}} = 1}}^{{\text{n}}} {T{}_{{\text{r}}}^{{\text{i}}} } \times C{}_{e}^{{\text{i}}} /C{}_{{\text{n}}}^{{\text{i}}}$$

In the formula, $C{}_{{\text{f}}}^{{\text{i}}}$ is the pollution parameter of a certain metal, $C{}_{e}^{{\text{i}}}$ is the measured content of heavy metals in the environment, $C{}_{{\text{n}}}^{{\text{i}}}$ is the required reference value for calculation, $E{}_{{\text{r}}}^{{\text{i}}}$ is the potential ecological risk parameter of a certain metal, $T{}_{{\text{r}}}^{{\text{i}}}$ is the toxicity response coefficient of a single pollutant, and RI is the potential ecological risk index of multiple metals. The pollution level is shown in Table 4

**Table 4:** Classification criteria of E_r_ and RI.

$E{}_{{\text{r}}}$	RI	Pollution level
≤ 40	≤ 150	Mild ecological hazards
40–79	150–299	Moderate ecological hazards
80–159	300–600	Strong ecological hazards
160–320	≥ 600	Very strong ecological hazard
≥ 320		Extremely strong ecological hazards

The background values were selected from the background values of Zhejiang Province in "Background Values of Soil Elements in China^31–34^ as the basis, as shown in Table 5.

**Table 5:** Background values of soil elements in Zhejiang Province^15^(mg/kg soil).

Heavy metal	Cd	As	Pb	Cr	Cu	Zn
Background Level	0.07	9.20	23.70	52.9	17.60	70.6

### Result and discussion

#### Current situation and source analysis of heavy metal pollution

**Figure 2:**
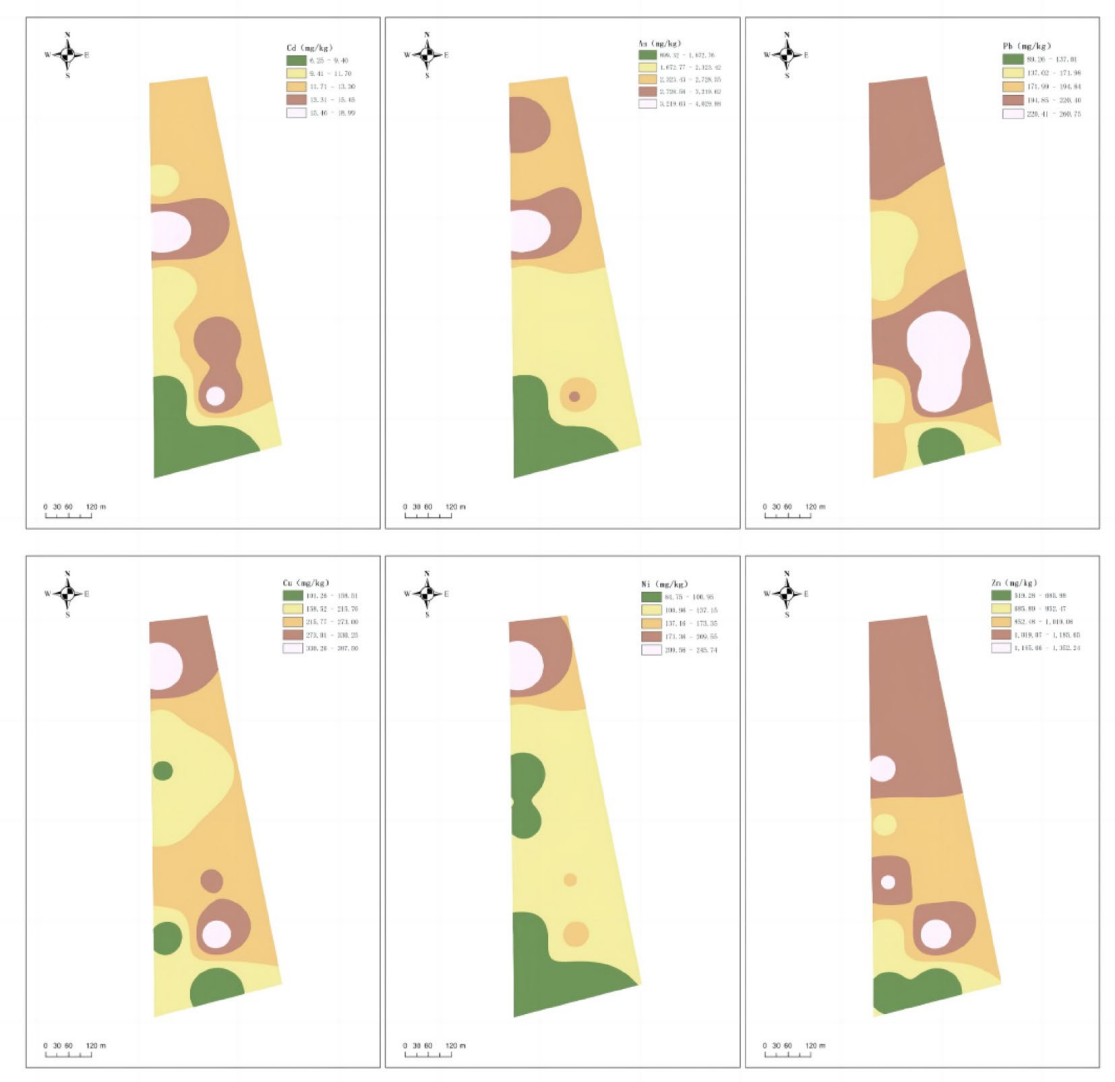
Heavy metal content in different soil layer (mg/kg).

The survey was conducted on the downwind soil around a metal surface treatment company in Jiaxing City, which was collected in three layers, and a total of 30 sampling data were analyzed regionally. According to the average value of the soil in Hangzhou–Jiaxing–Huzhou Plain as the background value and the standard values of various elements specified in the secondary standard of the Soil Environmental Quality Standard GB 15,618–1995 (pH < 6.5)^35–38^, the pollution degree of heavy metals can be simply and intuitively displayed by using the multiple of exceeding the standard, The coefficient of variation reflects the interference of human activities on heavy metal content, and the larger the coefficient of variation, the stronger the interference from human activities. To investigate the overall pollution status of heavy metals around Jiaxing Metal Surface Treatment Company and the impact of its location and distance from the pollution source, the dominant wind direction in Jiaxing is southeast wind. This study measured the average content of heavy metals in the downwind direction of the area and the content of heavy metals at three different sampling depths. The results are shown in Table 6 and Fig. 2.

**Table 6:** The heavy metal content in surface (0–30 cm).

Heavy metal	Detection range (mg/kg)	Average value (mg/kg)	Coefficient of variation (%)	Average number of exceedances	Average exceedance rate (%)	Standard value (mg/kg)
Cd	1.25–19	8.583	0.51	14.31	100	0.6
As	229–4030	1560.6	0.58	62.424	100	25
Pb	89.25–300	193.35	0.27	0	86.6	140
Cr	121.5–269.75	200.67	0.23	0	0	300
Cu	101.25–1440.75	263.26	0.92	0	43.3	200
Ni	64–289.5	127.23	0.43	0	60	100
Zn	339.25–1391.75	848.32	0.38	0	100	250

From Table 6, it can be seen that the coefficient of variation reflects the impact of human activities on heavy metal content, with a coefficient of variation of 92% for Cu, indicating that the Cu content around the factory area has been severely affected by human activities. Secondly, the coefficient of variation of As reached 58%, which may be due to the use of arsenic containing fertilizers and pesticides causing metal residues^35^. The coefficient of variation of Cd and Ni reached 51% and 43% respectively, mainly considering the dry and wet deposition of Cd or Ni into the soil caused by industrial waste gas^35^. The six heavy metals in the surface soil generally exceed the standard, with Zn, Cd, and As exceeding 100%, Pb exceeding 86.6%, and Cu having the lowest exceeding rate of 43.3%. The pollution of zinc and arsenic is extremely severe and unevenly distributed, while the distribution of lead, cadmium, nickel, and chromium is relatively uniform.

Research has shown that heavy metals in soil are not only related to species, but also to the relative location and distance of pollution sources^5^. Zhao Renxin et al.^34^ concluded from their research on Inner Mongolia that the influence of wind direction on the distribution of heavy metals is not significant. The main reasons for this phenomenon may be three aspects: interference from other enterprises' emissions; The interference of agricultural pesticides. Atmospheric sedimentation caused by wind power; The airflow disturbance caused by vehicles traveling to the west of the factory area weakens the role of natural wind direction. In this study, the content of As in all directions and locations exceeded the background value of soil in Jiaxing City^6^, and the content of As in the 0–10 cm soil surface reached the maximum value of 4030 mg/kg, which exceeded the standard by 62.42 times. This may be caused by the disordered discharge of arsenic containing wastewater and the atmospheric sedimentation of waste gas generated during the electroplating production process of the factory. In addition, the use of organic arsenic pesticides in the surrounding areas has also caused a large amount of arsenic residue. Long term inhalation or oral administration of small amounts of arsenic containing steam can lead to chronic poisoning. Arsenic pollution can cause toxicity and reproductive harm and skin diseases, gastrointestinal tract, liver and kidney, cardiovascular system, and other cancers, such as skin cancer and lung cancer. Effective measures must be taken to prevent and treat it. Except for As, Cd pollution exhibits the same distribution pattern as As, reaching its peak at the surface layer of 0–10 cm soil. Cd can enter the human body through the food chain and accumulate in the body, causing chronic poisoning, liver and kidney damage, and bone metabolism obstruction^25^, therefore special attention is also needed. The exceedance rate of Pb may be due to its proximity to highways, automobile exhaust emissions, tire wear, and corporate exhaust emissions, which exceed the standard. Cr has the lowest exceeding standard rate and average exceeding standard multiple among the 7 tested heavy metals, which may be related to the layout of drainage channels.

### Index of geo-accumulation

**Figure 3:**
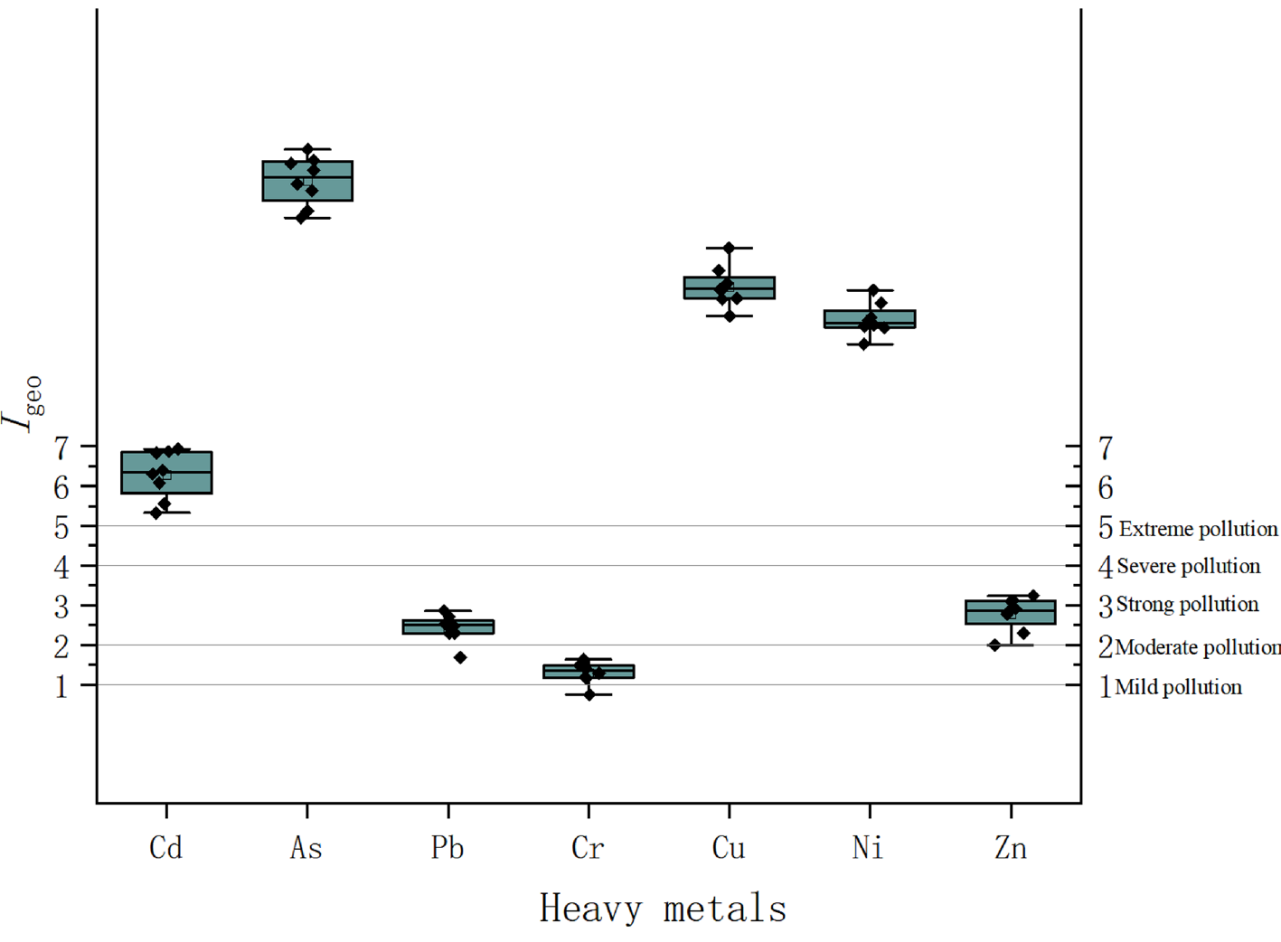
Soil heavy metal cumulative index vertical distribution of the study area.

The overall evaluation of six heavy metals in the soil around the metal factory using the land accumulation index method and the evaluation results of each metal at different vertical depths are shown in Fig. 3.

From Fig. 3, it can be seen that the surface soil (0–10 cm) has a high ground accumulation index, indicating that the surface soil has also been severely polluted. The ground accumulation index of the middle soil (10–20 cm) is mostly at a high level, which may be the result of downward infiltration of the surface layer. The ground accumulation index of As is the highest, reaching 6.67. All soils are polluted to varying degrees by As, mostly concentrated at extremely severe pollution levels, and its severity is similar to each soil layer. Secondly, both the surface and deep soils of Cu and Ni are severely polluted. The overall pollution of Zn and Pb is between moderate to strong pollution. Compared with other heavy metals, Cr has the lightest pollution level, but still reaches a moderate level of pollution. Out of the 7 heavy metals measured in this study, 6 were found to have moderate or higher levels of pollution. Considering their potential harm to surrounding residents, three heavy metals, As, Zn, and Cd, were selected as risk factors for human health risk assessment.

### Ecological risk assessment results and analysis

After a series of data processing, we obtained the following results as shown in Table 7

**Table 7:** Ecological assessment results.

Heavy metal	Cd	As	Pb	Cr	Cu	Zn
Background value($C_{n}^{i}$)	0.07	9.20	23.70	52.9	17.60	70.6
Average ($C_{s}^{{\text{i}}}$)	8.58	1560.6	193.35	200.67	263.26	848.32
Pollution parameters($C_{{\text{f}}}^{{\text{i}}}$)	0.60	14,357.52	4582.4	10,615.44	4633.38	59,891.39
Toxicity coefficient of heavy metals($T_{r}^{1}$)	30	10	5	2	5	1
Ecological hazard coefficient ($E_{{\text{r}}}^{{\text{i}}}$)	18.02	143,575.2	22,911.98	21,230.89	23,166.88	59,891.39
Multiple heavy metals (RI )	270,794.351					

After ecological risk assessment, we found that the heavy metals in the soil around Junchi Electroplating Plant in Jiaxing City belong to extremely strong ecological hazards. Except that the ecological hazard index of cadmium is only 18.02, which belongs to mild ecological hazards, the other six heavy metals are extremely harmful, especially arsenic, which reaches 143,575.2. Next, we should pay more attention to soil remediation.

### Health risk assessment

#### Exposure assessment analysis

In this study, non-carcinogenic daily exposure assessment of three heavy metals, As, Zn, and Cd, to children and adults was carried out, and the results are shown in Table 8.

**Table 8:** Daily exposure doses of heavy metals in soil mg/(kg d).

		*EDI*_breathe_		*EDI*_skin_		*EDI*_*mouth*_		*EDI*_total_	
Metals	Classification	Children	Adult	Children	Adult	Children	Adult	Children	Adult
As	Maximum	1.15E–07	3.27E–07	2.33E–06	2.96E–05	4.17E–03	2.96E–03	4.17E–03	2.99E–03
	Minimum	6.53E–09	1.86E–08	1.33E–07	1.68E–06	2.37E–04	1.68E–04	2.37E–04	1.70E–04
	Average	4.45E–08	1.27E–07	9.04E–07	1.15E–05	1.61E–03	1.15E–03	1.61E–03	1.16E–03
Cd	Maximum	5.42E–10	1.54E–09	1.10E–08	1.40E–07	1.96E–05	1.40E–05	1.97E–05	1.41E–05
	Minimum	3.56E–11	1.01E–10	7.24E–10	9.19E–09	1.29E–06	9.19E–07	1.29E–06	9.28E–07
	Average	2.45E–10	6.96E–10	4.97E–09	6.31E–08	8.87E–06	6.31E–06	8.88E–06	6.37E–06
Zn	Maximum	8.25E–09	2.35E–08	1.68E–07	2.13E–06	2.99E–04	2.13E–04	2.99E–04	2.15E–04
	Minimum	1.82E–09	5.19E–09	3.71E–08	4.71E–07	6.62E–05	4.71E–05	6.62E–05	4.75E–05
	Average	3.63E–09	1.03E–08	7.37E–08	9.35E–07	1.32E–04	9.35E–05	1.32E–04	9.45E–05

From Table 8, it can be seen that the oral intake of heavy metals is much higher than the heavy metal content through skin contact and respiratory inhalation. The daily intake of heavy metals in soil through the three pathways is in the order of EDI _oral_ > EDI _skin_ > EDI _respiratory_. As children's oral intake of heavy metals is higher than that of adults, but the heavy metal content through skin contact and respiratory inhalation is lower than that of adults. The exposure dose reaches its maximum when children ingest As element through oral intake, which is 4.17 × 10^−3^mg/(kg/d), the minimum exposure dose appears in children who inhale Cd through breathing, with a minimum value of 3.56 × 10^-11^mg/(kg/d). The doses of other metals Zn and Cd ingested by mouth, skin contact, and respiratory inhalation in adults are greater than those in children.

#### Health risk assessment

The non carcinogenic and carcinogenic risk assessment indices of As, Zn, and Cd heavy metals on human health are shown in Table 9. From Table 9, it can be seen that the non carcinogenic risk index (HQi) of various pathways around a heavy metal surface treatment plant in Jiaxing is partially greater than 1. Relatively speaking, the risk of oral intake is the highest, while the non carcinogenic risk of direct skin contact and respiratory inhalation is relatively small, The results are consistent with the health risk assessment conclusion of heavy metals in subway station dust studied by Yang Xiaozhi^39^. The maximum occurrence of HQi is in children's oral intake of heavy metal As, with a maximum value of 12.34; The minimum value is the respiratory intake of heavy metal Zn by children, with a minimum value of 7.48 × 10^–6^. In addition, the dose of Cd for oral intake, skin contact and inhalation of adults is higher than that of children, which is consistent with the conclusion of Exposure assessment, indicating that the non carcinogenic risk index is related to the exposure route. Regardless of the exposure pathway, the non carcinogenic health risk assessment of As is higher than that of the other two heavy metals, therefore, the maximum non carcinogenic health risk hazard of As is 12. In addition, the total non carcinogenic health risk HQ of Cd and Zn for adults and children is less than 1, indicating that these elements do not pose a non carcinogenic health risk to residents around the factory area. When Guo Pengran et al. studied the soil pollution situation around electroplating plants^40^, they also found that the carcinogenic risk of As and Cr in the soil was greater than 10^–4^, which was higher than the maximum acceptable risk level, similar to the results of this study.

**Table 9:** The index of health risk.

Risk index	Classification	As	Cd	Zn
HQ_mouth_	Child	1.23E + 01	1.55E–03	3.61E–03
	Adult	6.02E + 00	2.57E–03	5.99E–03
HQ_Breathe_	Child	7.11E–01	1.14E–05	2.58E–06
	Adult	1.18E + 00	1.89E–05	4.27E–06
HQ_skin_	Child	6.91E–03	8.67E–04	7.48E–06
	Adult	4.10E–02	5.14E–03	4.44E–05
HI_total_	Child	1.31E + 01	2.43E–03	3.62E–03
	Adult	7.24E + 00	7.73E–03	6.04E–03
Risk_mouth_	Child	2.42E–03	3.37E–06	1.10E–04
	Adult	1.72E–03	2.40E–06	7.86E–05
Risk_breathe_	Child	6.67E–08	9.29E–11	3.05E–09
	Adult	1.90E–07	2.64E–10	8.67E–09
Risk_skin_	Child	1.36E–06	1.89E–09	6.19E–08
	Adult	1.72E–05	2.40E–08	7.86E–07
Risk_total_	Child	2.42E–03	3.37E–06	1.11E–04
	Adult	1.74E–03	2.42E–06	7.94E–05

The maximum values of As's Carcinogenic Health Risk Index (RISK) for adults and children appear under the exposure pathway of oral intake, with values of 1.72 × 10^–3^ and 2.42 × 10^–3^, there is a high risk of cancer. The carcinogenic risk of Cd is consistent with that of As, with its maximum values occurring through oral intake in children, at 3.37 × 10^–6^. The minimum reference risk index for carcinogenicity of Cd is 9.29 × 10^–11^, occurring in children through respiratory exposure pathways, within an acceptable range of carcinogenic risk. Overall, the total cancer risk index of As through three pathways is relatively high, and children are higher than adults, which should be highly valued and strengthened for prevention and control; The excessive levels of As may be related to the application of pesticides in farmland or to nearby agricultural companies^21^. Zn does not have a carcinogenic risk. The carcinogenic health risk index for adult oral intake is 1.10 × 10^–4^, which has exceeded the warning value, if not controlled, it will pose a threat to the health of surrounding residents.

## Conclution

1. After processing the soil environment sampling data around Junchi Electroplating Plant in Jiaxing this time, we found that the surrounding soil was affected to varying degrees. According to the southeast wind of Jiaxing City, the average exceeding standard rate of the surrounding heavy metals cadmium and arsenic was 100%, and the average exceeding standard times of the heavy metals cadmium and arsenic were 14.31 and 64.42 respectively, far exceeding the content of other heavy metals. Therefore, we paid special attention to the remediation of these two heavy metals in the later stage.
2. According to the calculation of the ground accumulation index, several heavy metals are sorted: arsenic > cadmium > copper > zinc > lead > nickel. Arsenic and cadmium are evaluated as extremely heavy metal pollution, while copper and zinc are highly polluted. After seeing this result, we should pay more attention to the soil heavy metal problem around Junchi Electroplating Plant. Soil pollution should be controlled and repaired to ensure the safety of surrounding residents.
3. In the exposure risk assessment of this area, the value of consumption is much greater than the exposure risk harm caused by breathing and skin, with the maximum value of As exposure harm for children and adults being 4.17 × 10^–3^, in terms of cancer risk, the value brought by consumption is much greater than the respiratory and skin cancer risk index. Among the three heavy metals Cd, As, and Zn, their highest cancer risk score is 3.37 × 10^–6^, 2.42 × 10^–3^, 1.10 × 10^–4^. But among Cd, As, and Zn, the ones with the highest risk of cancer are consumed by children, adults, and children, respectively. Therefore, more attention should be paid to the soil environment around Jiaxing Junchi Electroplating Plant.

## Data Availability

The datasets used and analysed during the current study available from the corresponding author on reasonable request. All data generated or analysed during this study are included in this published article and its supplementary.
